# Systematic Epstein-Barr virus-positive T-cell lymphoproliferative disease presenting as a persistent fever and cough: a case report

**DOI:** 10.1186/1752-1947-8-288

**Published:** 2014-08-27

**Authors:** Fereshteh Ameli, Firouzeh Ghafourian, Noraidah Masir

**Affiliations:** 1 Department of Pathology, Faculty of Medicine, Universiti Kebangsaan Malaysia Medical Centre, Jalan Yaacob Latif, Bandar Tun Razak 56000, Cheras, Kuala Lumpur, Malaysia

**Keywords:** Epstein-Barr virus, T-cell lymphoproliferative disease, Childhood

## Abstract

**Introduction:**

Systemic Epstein-Barr virus-positive T-cell lymphoproliferative childhood disease is an extremely rare disorder and classically arises following primary acute or chronic active Epstein-Barr virus infection. It is characterized by clonal proliferation of Epstein-Barr virus-infected T-cells with an activated cytotoxic phenotype. This disease has a rapid clinical course and is more frequent in Asia and South America, with relatively few cases being reported in Western countries. The clinical and pathological features of the disease overlap with other conditions including infectious mononucleosis, chronic active Epstein-Barr virus infection, hemophagocytic lymphohistiocytosis and natural killer cell malignancies. We describe the rare case of systemic Epstein-Barr virus-positive T-cell lymphoproliferative childhood disease in a 16-year-old Malay boy.

**Case presentation:**

He presented with a six-month history of fever and cough, with pulmonary and mediastinal lymphadenopathy and severe pancytopenia. Medium- to large-sized, CD8+ and Epstein-Barr virus-encoded RNA-positive atypical lymphoid cells were present in the bone marrow aspirate. He subsequently developed fatal virus-associated hemophagocytic syndrome and died due to sepsis and multiorgan failure.

**Conclusions:**

Although systemic Epstein-Barr virus-positive T-cell lymphoproliferative childhood disease is a disorder which is rarely encountered in clinical practice, our case report underlines the importance of a comprehensive diagnostic approach in the management of this disease. A high level of awareness of the disease throughout the diagnosis process for young patients who present with systemic illness and hemophagocytic syndrome may be of great help for the clinical diagnosis of this disease.

## Introduction

Epstein-Barr virus (EBV) infection is asymptomatic in more than 90% of humans. However, in a minority, the infection is associated with a number of malignancies involving B cells, T cells, natural killer (NK) cells and epithelial cells [1].

EBV is considered to be an oncogenic virus and belongs to the herpes virus family. EBV infection is frequently associated with various lymphoproliferative disorders (LPDs). The relative frequency of LPDs, clinical presentation and EBV-positivity in different histological subtypes in immunocompetent hosts were found to vary in different geographical areas. This variation may be ascribed to genetic and environmental etiologic factors [2]. Hodgkin’s lymphoma (HD) and infectious mononucleosis are the most common EBV-associated diseases in Western countries, whereas in Asian and Latin American countries other EBV-associated LPDs, mainly those associated with T lymphocytes or natural killer (NK) cells, are more prevalent [2].

Different terms are assigned to EBV-associated T- or NK-cell LPDs based on their clinical findings and the immunophenotype of the proliferating cells. Some of these terms include T- or NK-cell-type chronic active EBV (CAEBV) infection, systemic EBV-positive T-cell lymphoproliferative childhood disease, hydroa vacciniforme-like lymphoma, aggressive NK-cell leukemia (ANKL), nasal type NK- or T-cell lymphomas, extranodal NK- or T-cell lymphoma and angioimmunoblastic T-cell lymphoma (with EBV in B cells).

A newly recognized EBV-associated malignancy is the systemic EBV-positive T-cell lymphoproliferative childhood disease. It is characterized by a life-threatening illness of children and young adults with clonal proliferation of EBV-infected T cells with an activated cytotoxic phenotype. It can develop either after primary EBV infection or related to chronic active EBV infection (CAEBV) [3].

These neoplastic T cells are usually small and lack significant cytological atypia. However, cases with pleomorphic medium- to large-sized lymphoid cells with irregular nuclei and frequent mitoses have been described. The most common typical phenotypes are CD2+, CD3+, CD56- and TIA+. Most of the cases that are secondary to primary EBV infection are CD8+, while the forms that occur in the setting of severe CAEBV are CD4+. Neoplastic cells have monoclonally rearranged T-cell receptor (*TCR*) genes, and consistent Epstein-Barr virus-encoded RNA (EBER)-positivity at *in situ* hybridization (ISH) [4].

In this report, we describe the clinicopathological findings of a fulminant systemic EBV-positive T-cell lymphoproliferative childhood disease in a 16-year-old Malay boy who, to the best of our knowledge, represents the first reported case of this disease in Malaysia.

## Case presentation

A 16-year-old Malay boy first presented with a history of non-productive cough and persistent fever for the past month. There was no associated weight loss, loss of appetite or other significant clinical findings at initial presentation. The family history was unremarkable. The fever was resistant to conventional therapies, and serial investigations could not determine the source of the suspected infection. He was hospitalized at three different governmental and private hospitals during the first three months and eventually, when a computed tomography (CT) scan of the thorax was performed, a solitary nodule at the base of left lung, measuring 12×11mm, was detected.

He was empirically treated for tuberculosis (ethambutol, isoniazid, rifampicin and pyrazinamide) but showed no sign of improvement after one month. He declined a CT-guided biopsy of the lung nodule and tuberculosis treatment was continued for three months. After one month of treatment he developed pancytopenia but declined a bone marrow biopsy. A subsequent CT scan of the thorax and abdomen (four months after the first admission) showed further enlargement of the lung nodule (32×31mm) with multiple new lesions appearing in both lungs, measuring between 5 and 11mm in diameter. In addition, there was an anterior mediastinal mass measuring 26×46×40mm. At this point a hematological malignancy or severe fungal infection was suspected.

He refused medical advice and discharged himself from the hospital with no further treatment. Two months later he was readmitted with persistent fever and diarrhea, at which point he was referred to our institution for further management. He was found to be dehydrated and cachectic. His lung sounds were reduced basally on auscultation with bilateral crepitations. A hepatosplenomegaly was observed in the physical examination but no lymphadenopathy was found. A full blood picture showed severe pancytopenia with a hemoglobin level of 5.1g/dL, a total white blood cell count of 0.6×10^9^/L (neutrophils 0.1×10^9^/L and lymphocytes 0.5×10^9^/L) and platelet count of 57×10^9^/L. His lactate dehydrogenase (LDH) was elevated (550U/mg) and his blood culture, rheumatoid factor, antinuclear antibody, leptospirosis serology, blood film for malaria parasite and Monospot tests were negative for EBV. A repeat CT scan revealed similar lung and mediastinal lesions as well as hepatosplenomegaly and right iliac lymphadenopathy.

**Figure 1:**
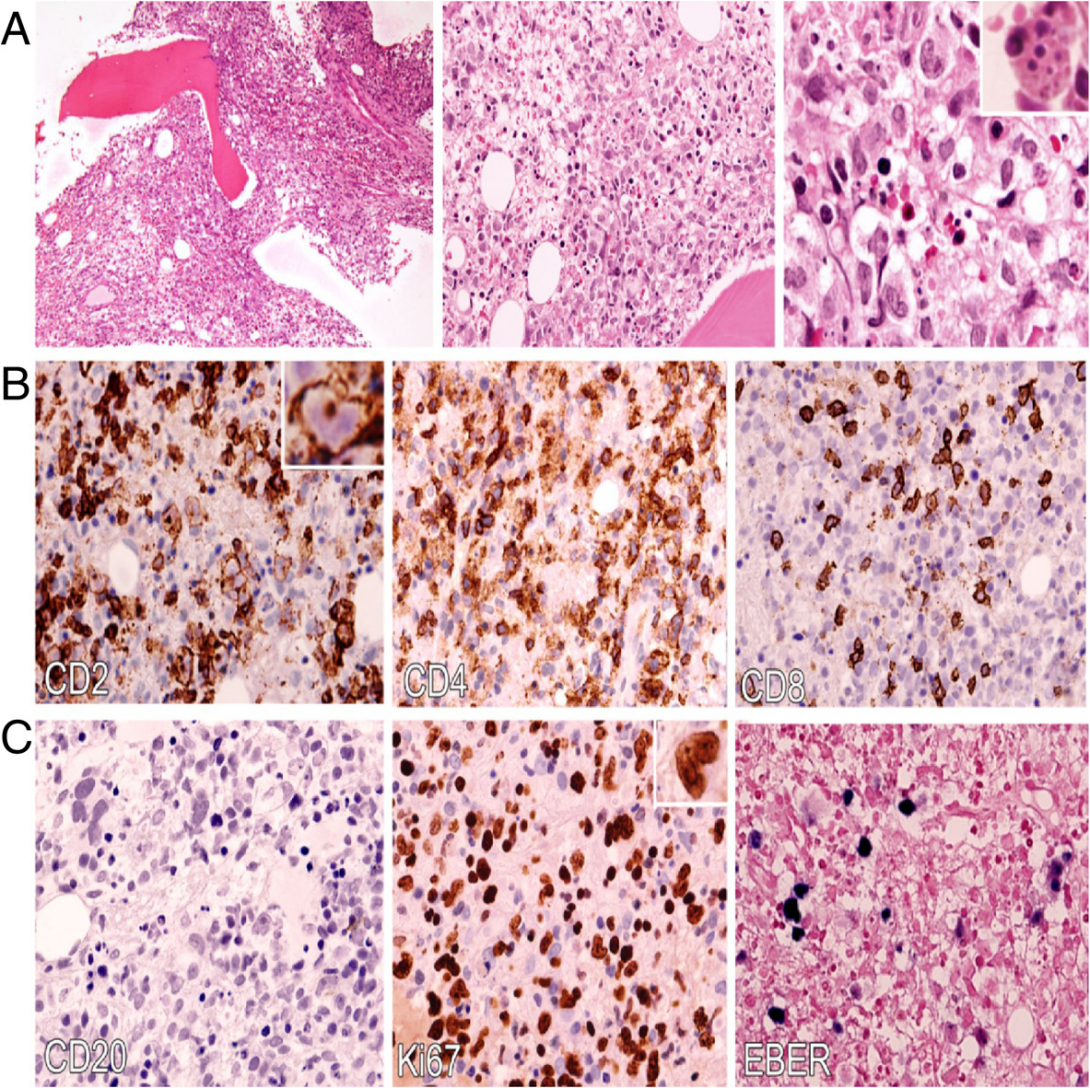
**Systemic Epstein-Barr virus-positive T-cell lymphoproliferative childhood disease: bone marrow biopsy. A**: Hematoxylin and eosin (H&E) sections illustrate the medium-sized lymphoid cells replacing the normal hematopoietic elements and exhibiting moderate atypia with irregular kidney-shaped nuclei, dispersed chromatin and distinct nucleoli (100x, 400x and 600x respectively left to right). Hemophagocytosis is also easily seen (inset). **B**: An immunohistochemical study shows that the majority of infiltrated atypical lymphocytes expressed CD2+ and CD4+ with scattered positivity to CD8. **C**. The atypical lymphoid cells are not immunoreactive to CD20 and there is a paucity of B cells in the bone marrow. Ki-67 immunostaining shows a high proliferative index and EBV-encoded RNA (EBER) by *in situ* hybridization demonstrates a strong reactivity of atypical lymphoid cells.

A bone marrow biopsy was performed. The aspirate showed several clusters of abnormal lymphoid cells and hemophagocytic activity but assessment was limited due to hemodilution. Accordingly, he underwent treatment for hemophagocytic lymphohistiocytosis (HLH) (with unknown cause) based on the HLH-2004 protocol. This protocole was developed by the treatment protocol of the second international HLH study 2004 [5]. Immunophenotyping of the atypical lymphoid cells by flow cytometry was inconclusive due to suboptimum aspirate sampling. The trephine biopsy was hypocellular with a marked reduction of normal hematopoietic elements. This was replaced by scattered medium- to large-sized malignant lymphoid cells. The cells were moderately pleomorphic with a kidney-shaped nuclei, irregular nuclear membrane and prominent nucleoli (Figure 1). Occasional mitotic figures, including abnormal forms and hemophagocytosis, were also noted. It was revealed upon immunohistochemistry that the atypical lymphoid cells expressed CD3, CD2 and CD4 positivity (Figure 1). A few atypical cells were also positive for CD8. The malignant cells were negative for CD20, CD56 and ALK1 (Figure 1). Ki-67 immunostaining highlighted an increased proliferative rate in the malignant cells. *In situ* hybridization for EBV encoded RNA (EBER) demonstrated EBV-positive atypical lymphoid cells (Figure 1). Therefore a diagnosis of systemic EBV-positive T-cell lymphoproliferative childhood disease was made.

Unfortunately his health status rapidly deteriorated and he developed multiorgan failure, including oliguric acute renal failure, pericarditis, acute hepatitis and disseminated intravascular coagulation. He subsequently presented with generalized tonic-clonic seizures and a further CT scan revealed an acute left frontal subarachnoid haemorrhage. He died 15 days after admission to our center.

## Discussion

Two major types of Epstein-Barr (EBV) associated T-cell lymphoproliferative disorders have been reported in the pediatric age group. Both types have higher frequency in Asians and Native Americans (Central and South America and Mexico) [4]. Hydro vacciniforme-like lymphoma is a cutaneous malignancy which presents with a papulovesicular eruption in sun-exposed skin. This disease is associated with ulceration and an indolent clinical course which usually progresses over time. Systemic EBV-positive T-cell lymphoproliferative childhood disease may occur shortly after primary acute EBV infection or in the setting of chronic active EBV infection (CAEBV). It has a rapid progression and can result in multiple organ failure, sepsis and death within days to weeks [4].

In normal individuals, primary EBV infection of B cells causes B-cell proliferation and a robust cytotoxic T-cell response that explains the lymphadenopathy, splenomegaly and circulating atypical CD8+ T cells, which are characteristic of infectious mononucleosis. The emergence of EBV-specific cytotoxic T cells suppresses the proliferative effects of EBV, and the virus persists in a dormant form in resting memory B cells of normal carriers. When functioning EBV-specific cytotoxic T cells cannot be generated, T cells become infected by the virus and therefore fatal infectious mononucleosis or CAEBV occurs. EBV-positive T cells may also clonally expand and develop an overt T-cell lymphoma [6].

Several clinical disorders have overlapping features with systemic EBV-positive T-cell lymphoproliferative childhood disease and are now incorporated into the latter according to the revised 2008 World Health Organization (WHO) classification [4]. These include EBV-associated hemophagocytic syndrome, fatal infectious mononucleosis, viral-associated hemophagocytic syndrome, severe CAEBV, EBV-associated T- or NK-cell LPD, and fulminant EBV-associated T-cell lymphoproliferative disorder after acute or chronic EBV infection [7]. The WHO classification (2008) requires evidence of systemic illness and T-cell monoclonality for the diagnosis of systemic EBV-positive T-cell lymphoproliferative childhood disease [6].

The T-cell proliferation in systemic EBV-positive T-cell lymphoproliferative childhood disease is monoclonal. Notably, CD8+ is seen in cases arising following primary EBV infections, whereas cases arising in the setting of severe CAEBV are CD4+. Co-expression of CD4 and CD8, as shown in our case report, has been previously reported [7].

Interestingly, the relative paucity of B cells is a consistent bone marrow finding in systemic the disease, which is contrary to the frequent mature B cells and B-cell precursors in normal pediatric bone marrow. The presence of an EBV-positive T-cell infiltrate with a paucity of B cells, as seen in this case, may serve as a clue to the diagnosis of systemic EBV-positive T-cell lymphoproliferative childhood disease [6].

The differential diagnosis of systemic EBV-positive T-cell lymphoproliferative childhood disease includes primarily extranodal NK- or T-cell lymphoma, nasal-type (NKTCL-NT), aggressive NK-cell leukemia and fulminant infectious mononucleosis. The distinguishing clinical presentation is the immunophenotype as well as the identification of clonal *TCR* gene rearrangement which can differentiate this disorder from NKTCL-NT. NK-cell leukemia and systemic EBV-positive T-cell lymphoproliferative childhood disease have many common clinicopathologic features, but unlike the EBV-positive T-cell lymphoproliferative childhood disease, NK-cell leukemia is not preceded by primary EBV infection or CAEBV, and the *TCR* gene is in the germ line configuration. Although it might be difficult to differentiate fulminant infectious mononucleosis from systemic EBV-positive T-cell lymphoproliferative childhood disease due to the overlapping clinical and morphologic features, EBV-positive T-cell lymphoproliferative childhood disease is characterized by a polyclonal EBV-positive B-cell proliferation with a predominance of immunoblasts and plasma cells [7].

Our review of previously reported cases of acute T-cell LPD following primary infection with EBV showed that most of the reported cases were from Asia [4]. It has been hypothesized that the high prevalence of EBV-positive T-cell lymphoproliferative childhood disease among Asians and Mexicans may be due to a genetically determined susceptibility due to the existence of certain human leukocyte antigen (HLA) types which result in an abnormal response to EBV infection [8].

In a study by Yoshii *et al.* 17 cases of systemic EBV-positive T-cell lymphoproliferative childhood disease following acute EBV infection were reviewed [9]. They found a higher incidence of the disease in boys (boy to girl ratio, 11:6), as opposed to the recent WHO 2008 classification which reported no such predisposition [4]. The patient in our case report was a boy, in agreement with the findings of Yoshii *et al.*[9].

Prolonged fever of unknown origin preceding the diagnosis is a characteristic clinical finding. As observed in this case, the accompanied cough resulted in an initial error in diagnosis and treatment for tuberculosis. The underling cause of cough in this case could be due to lung and mediastinal masses that were noted in the CT scan. In all previously reported cases, the clinical behaviour was aggressive and showed poor response to intensive chemotherapy and supportive care. Death usually occurs between days and 13 months (mean, 33 days) of initial diagnosis [8]. In this case, death occurred six months after presentation. Sepsis and multiorgan failure were reported to be the most frequent causes of death, which is similar to the case presented here [5,7,8].

The liver and spleen are the most common sites of involvement, followed by lymph nodes, bone marrow, skin and lung [4]. Our patient presented with lung mass and mediastinal lymphadenopathy but unfortunately, a biopsy from lung and mediastinal lesions was not performed because of family refusal.

A relatively common finding in most of the patients in previous reports was the lack of elevated antibody titers or, in some cases, the total absence of serologic responses to EBV. In this case, a serologic study for EBV was not performed due to a lack of suspicion of EBV during the diagnosis process. EBV serology may be clinically misleading since it does not suggest acute primary or active infection. This apparent lack of humoral response to the virus may increase the risk of T-cell infection and subsequent monoclonal expansion [9].

Patients with hemophagocytic syndrome were shown to have elevated levels of cytokines in the serum [10]. The released cytokine causes the clinicopathological manifestations of the hemophagocytic syndrome, as was seen in our patient. The sources of cytokines may be activated T cells or macrophages [10].

No effective treatment has been suggested for systemic EBV-positive T-cell lymphoproliferative childhood disease although antiviral therapy, immunosuppressive agents and chemotherapy can delay disease progression. A hematopoietic stem-cell transplant is the only curative option.

## Conclusions

In summary, we reviewed the clinicopathological features of systemic EBV-positive T cell lymphoproliferative childhood disease following acute EBV infection to facilitate our diagnosis. Our case report implies that clinical awareness of the disease, accompanied with an immunohistochemical study and EBER *in situ* hybridization could be useful for diagnosis of the disease in developing countries where molecular studies are not necessarily available.

Although no specific treatment is available at present, prompt attempts to control the hemophagocytic syndrome and rapid administration of anti-Epstein-Barr virus treatment are recommended.

Since the clinical progression of systemic EBV-positive T-cell lymphoproliferative childhood disease is aggressive, additional studies and case reviews are useful in determining early and accurate diagnostic and therapeutic strategies for this disease.

## Consent

Written informed consent was obtained from the patient’s parents for publication of this case report. A copy of the written consent is available for review by the Editor-in-Chief of this journal.

## Competing interests

The authors declare that they have no competing interests.

## Authors’ contributions

FA analyzed and interpreted the patient data regarding the hematological disease and the transplant. FA, FG and NM performed the histological examination, and NM was a major contributor in writing the manuscript. All authors read and approved the final manuscript.
